# What Do We Demand? Responding to the Call for Precision and Definitional Agreement in Family Planning’s “Demand” and “Need” Jargon

**DOI:** 10.9745/GHSP-D-22-00030

**Published:** 2022-02-28

**Authors:** Madeleine Short Fabic

**Affiliations:** a Associate Editor, *Global Health: Science and Practice Journal*; Supervisory Public Health Advisor, U.S. Agency for International Development, Washington, DC, USA.

## Abstract

This commentary offers a response to the call to improve family planning language that describes “need” and “demand” and proposes a set of recommendations to add precision, improve measurement, and foster shared understanding in family planning.

See related article by Speizer et al.

## INTRODUCTION

Jargon—the specialized language used by people in the same work or profession—is only communicative if the people working in that profession have a common understanding of its meaning. As with most fields, family planning (FP) has its share of jargon and its own debates about the meaning of various pieces of jargon. As Speizer et al.^1^ describe in this issue of *GHSP,* 2 terms, in particular, are frequently misused and misinterpreted: “demand” and “need.” Speizer et. al.^1^ make a call to action for the broader FP community to improve language and metrics to describe demand and need and to ensure that “the perspectives of users, potential users, and nonusers are included.” This commentary offers an initial response to that call: it disambiguates several key terms; offers definitions for a set of interrelated yet distinct FP concepts; elaborates upon ongoing definitional and measurement challenges; and offers a set of recommendations to add precision, improve measurement, and foster shared understanding. Its main contribution is a demand typology framework, which can buttress existing, ongoing, and new efforts to respond to the call to action.

## DEFINING FP JARGON

One of the initial areas of confusion in FP jargon is the field’s frequent use of economic terms. Borrowed language includes “desire,” “want,” “need,” and “demand.” According to many economists,^2^^,^^3^ “desire” is a wish, and “want” is a nonessential desire. “Need” is a necessity, essential for life, and “demand” as a desire plus ability and willingness to enact that desire (Box 1). In the context of disambiguating FP’s “need” and “demand” terminology and measures, definitions are crucial.

One of the initial areas of confusion in FP jargon is the field’s frequent use of economic terms.

BOX 1 **Defining Economic Terms Adopted by Family Planning**

**Desire:** A wish.

**Want:** A nonessential desire.

**Need:** An essential desire, a necessity, essential for life.

**Demand:** Desire plus ability and willingness to enact that desire.

Also crucial is having a shared definition of “family planning.” FP generally supports the goal that anyone and everyone who has a desire to avoid pregnancy can be protected by voluntary, safe, and effective contraception (Box 2). FP is a broader concept than contraception, though like contraception, FP is generally focused on pregnancy prevention.^*^

BOX 2 **A Working Definition of “Family Planning”^a^**

**Family planning:** The services, policies, information, attitudes, practices, and commodities, including contraceptives, that give individuals who desire to avoid pregnancy the ability to do so.

^a^ Adapted from Starbird et al.^4^

Because FP is focused on meeting the pregnancy prevention needs of individuals who desire to limit or avoid pregnancy, FP aims to transform need into demand. In this context, “demand” means that individuals who express the need to prevent pregnancy are willing and able to achieve that need, typically by using modern contraceptive methods. FP then sets out to meet demand with adequate supply (i.e., accessible contraceptives).

Reflecting on definitions and meanings, using the term “need” here, rather than “want,” represents an important linguistic nuance. The need to avoid unwanted or unplanned pregnancy is essential. An individual’s ability to prevent pregnancy is not something “nice to have,” it is necessary—necessary for health, empowerment, education, wealth, and beyond.^4^

In addition to the need and demand to prevent pregnancy, there are other types of desires, wants, needs, and demands related to FP, including demand to have reproductive autonomy, to use contraception, and to choose a specific contraceptive method (Figure). These various types of desires and demands further complicate shared understanding of FP terms, and this lack of shared understanding distorts measurement approaches and interpretations. To add clarity, included herein is a brief description of each interrelated yet distinct type of FP-related desire and demand (Box 3).^5^

BOX 3 **Defining the 4 Types of Family Planning-Related Desires and Demands**

**Desire/Demand for Reproductive Autonomy**: “Having the power to decide and control contraceptive use, pregnancy, and childbearing. For example, people with reproductive autonomy can control whether and when to become pregnant, whether and when to use contraception, which method to use, and whether and when to continue a pregnancy.”^5^ As previously described, an individual’s desire for reproductive autonomy becomes demand when that individual is willing and able to achieve that desire. Uniquely, reproductive autonomy encompasses all other types of family-planning related demand.

**Desire/Demand to Delay or Limit Pregnancy**: In the case of desire, it is an individual’s stated preference to avoid pregnancy/childbearing in the near-term (i.e., delay, usually measured as within the next 1-2 years) and/or long-term (i.e., limit). An individual’s desire to limit or delay pregnancy transforms into demand when the individual is willing and able to enact that desire—through prolonged abstinence, breastfeeding, or contraception (modern or traditional).^a^

**Desire/Demand for Contraception**: Similar to, but not to be confused with “demand for family planning,”^b^ this concept refers to an individual’s desire to use a method or device that prevents pregnancy. Desire becomes demand when that individual is willing and able to use contraception.

**Desire/Demand for a Specific Contraceptive Method:** An individual’s desire for a specific contraceptive method. An individual’s desire transforms into demand when that individual is willing and able to use their preferred method.

^a^ Abortion serves to limit or delay childbearing but does not delay or limit pregnancy. If the focus were on the demand to delay or limit childbearing, rather than demand to delay or limit pregnancy, abortion would be included in this list.

^b^ “Demand for family planning” is a common family planning indicator. It is defined as the sum of (a) the number of women of reproductive age who are currently using (or whose partner is currently using) contraception and (b) the number of women of reproductive age who are classified as having unmet need for family planning.

**FIGURE:**
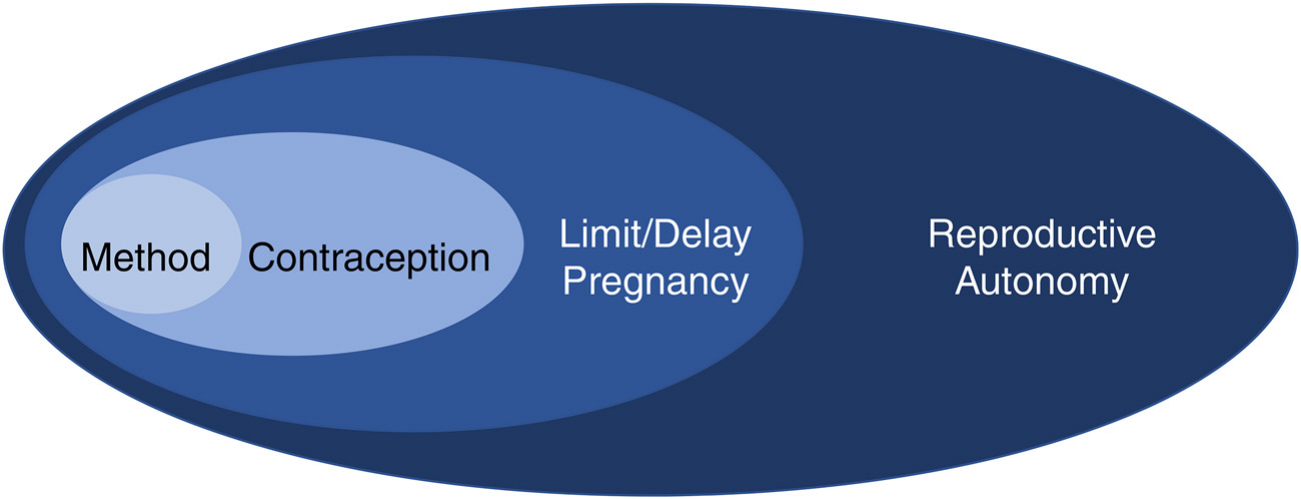
Four Types of Interrelated Yet Distinct Family Planning-Related Desires/Demands

## DEFINITIONAL AND MEASUREMENT CHALLENGES AND RECOMMENDATIONS

FP tends to conflate these 4 types of desires and demands, which creates some challenges for the field, especially for indicator development and measurement.

1. FP measurement usually categorizes an individual’s fertility desires as a binary (wants/doesn’t want; wants now/wants later). This concept is typically measured through direct questions to individuals about their fertility desires. For example, the Demographic and Health Surveys Program asks women aged 15–49 years, “Would you like to have (a/another) child, or would you prefer not to have any (more) children?” and “How long would you like to wait from now before the birth of (a/another) child?”^6^ However, fertility intentions are fluid.^7^ Additionally, many individuals express ambivalent or indifferent pregnancy intentions.^8^ As a result, the dichotomous approach to measuring an individual’s pregnancy prevention desires is overly simplistic and inaccurate.^9^ Pregnancy desires rest on a continuum and are perhaps better understood through the lens of pregnancy acceptability.^10^
2. FP characterizes individuals who express the need to limit or delay pregnancy, but who are lacking the willingness and/or ability to enact that need as having “unmet need for family planning” and having “demand for family planning.” These characterizations have several problems. First, “unmet need for family planning” is a misnomer. Based on a literal interpretation of needs expressed by individuals, the accurate framing is “unmet need for pregnancy prevention.” Second, the need for pregnancy prevention is not equivalent to the need for contraception. For example, pregnancy prevention methods include not only contraception (modern and traditional) but also prolonged abstinence and breastfeeding, however, FP characterizes individuals who are using prolonged abstinence or breastfeeding to prevent pregnancy as having “unmet need for family planning.”^11^^,^^12^ The field conflates the need for pregnancy prevention with the need for contraception. Again, the desire to limit or delay pregnancy is not equivalent to the desire to use contraception. Finally, characterizing individuals with the expressed need to prevent pregnancy as individuals with “demand for family planning” is incorrect. Demand is present when an individual has a willingness and ability to address their need. Individuals who express the need to prevent pregnancy do not necessarily have demand for FP.
3. FP assumes everyone who is using contraception has “met demand for family planning.” This assumption also has several problems. First, not all contraceptive users are using them for FP purposes. For example, individuals may demand contraception for nonpregnancy-related reasons, including to protect against sexually transmitted infections and/or for other health-related reasons, such as to reduce the severity of menstrual-related cramping and bleeding. Second, some individuals may be contraceptive users despite wanting to become pregnant. For example, individuals who have been unable to access implant removal services would be captured as having “met demand” when, in fact, they have no demand for contraception and their reproductive autonomy has been curtailed. Finally, some individuals may be dissatisfied with their contraceptive method. These individuals would be classified as having “met demand for family planning,” without regard to their desire for other contraceptive method(s).

Ideally, the FP field would aim to measure and understand all 4 types of interrelated, yet distinct desires, wants, needs, and demands. This requires more indicators that are more precise, which requires more complex data collection. To start, FP measurement can focus on answering a set of questions (Table) and developing a larger set of indicators that better address the 4 types of FP-related desires, wants, needs, and demands, among contraceptive users, potential users, and nonusers. Many of these questions have been addressed in recent years and related indicators have been proposed.^13^^–^^16^ Each of these efforts is represented in this proposed FP-related demand typology (Figure, Box 3, Table).

Ideally, the FP field would aim to measure and understand all 4 types of interrelated, yet distinct desires, wants, needs, and demands.

**TABLE.** Relevant Questions and Potential Indicators to Measure the 4 Interrelated Needs/Demands in Family Planning

Desire/Demand for	Relevant Questions to Address^a^	Potential Indicators
Reproductive autonomy	Does the individual believe it’s within their power to prevent pregnancy? Does the individual have the power to control their pregnancy prevention, contraception, and/or method use decisions?	In the context of family planning, a continuum based on: Individual’s perception of their power (self-efficacy) to prevent pregnancy Demand satisfied for pregnancy prevention Demand satisfied for contraception Demand satisfied for preferred contraceptive method
Pregnancy prevention	Does the individual express the need to prevent pregnancy? To what degree would the individual judge pregnancy as acceptable or unacceptable? Is the individual enacting that need (e.g., using contraception, prolonged abstinence, or breastfeeding)?	Among pregnant and non-pregnant individuals: Desire for pregnancy prevention Potential demand for contraception Demand satisfied for pregnancy prevention Unmet need for pregnancy prevention
Contraception	Does the individual express the desire to use contraception? Is the individual using contraception?	Among contraceptive users and non-users; users for family planning and users for non-family planning reasons: Desire for contraception Intention to use contraception Demand satisfied for contraception Unmet need for contraception Unmet need to discontinue contraception
Specific contraceptive method	Does the individual express the desire to use a specific form of contraception? Among nonpregnant individuals, is the individual using their preferred method of contraception?	Among contraceptive users/non-users: Desire for preferred contraceptive method Demand satisfied for preferred contraceptive method Unmet need for preferred contraceptive method

a These are not framed as survey questions.

By using shared definitions, recognizing a common demand typology, and addressing known measurement issues (language and beyond), FP can recapture shared meaning, understanding, and purpose. FP jargon can once again be communicative rather than obscuring.

## Footnotes

* Ideally, FP is more broadly focused on helping individuals achieve their fertility intentions, which includes not only pregnancy prevention but also pregnancy planning, infertility counseling, sexual and reproductive health education, body literacy, and more.

## Notes

**Peer Reviewed**

**First published online:** February 24, 2022.

## References

[1] Speizer IS Bremner J Farid S; FP2020 Performance, Monitoring, and Evidence Working Group. Language, use, and measuring contraceptive need and making these indicators more meaningful for measuring fertility intentions of women and girls. Glob Health Sci Pract. 2022;10(1):e2100450. 10.9745/GHSP-D-21-00450 PMC8885354 35294385

[2] Glossary of economics and personal finance terms. Federal Reserve Bank of Saint Louis. Accessed January 31, 2022. https://www.stlouisfed.org/education/glossary

[3] Roberts JL; World Health Organization Regional Office for Europe. *Terminology: A Glossary of Technical Terms on the Economics and Finance of Health Services.* WHO Regional Office for Europe; 1998. Accessed January 31, 2022. https://apps.who.int/iris/bitstream/handle/10665/108335/E69927.pdf

[4] Starbird E Norton M Marcus R. Investing in family planning: key to achieving the Sustainable Development Goals. Glob Health Sci Pract. 2016;4(2):191–210. 10.9745/GHSP-D-15-00374. 27353614 PMC4982245

[5] Measuring women’s reproductive autonomy. Bixby Center for Global Reproductive Health. March 17, 2014. Accessed January 31, 2022. https://bixbycenter.ucsf.edu/news/measuring-women%E2%80%99s-reproductive-autonomy

[6] The Demographic and Health Surveys Program. *DHS Model Questionnaire-Phase 8: Model Woman’s Questionnaire*. ICF; 2021. Accessed January 31, 2022. https://dhsprogram.com/pubs/pdf/DHSQ8/DHS8_Womans_QRE_EN_10Dec2021_DHSQ8.pdf

[7] Speizer IS Lance P. Fertility desires, family planning use and pregnancy experience: longitudinal examination of urban areas in three African countries. BMC Pregnancy Childbirth. 2015;15:294. 10.1186/s12884-015-0729-3. 26559486 PMC4641367

[8] Schwarz EB Lohr PA Gold MA Gerbert B. Prevalence and correlates of ambivalence towards pregnancy among nonpregnant women. Contraception. 2007;75(4):305–310. 10.1016/j.contraception.2006.12.002. 17362711

[9] Gómez AM Arteaga S Villaseñor E Arcara J Freihart B. The misclassification of ambivalence in pregnancy intentions: a mixed-methods analysis. Perspect Sex Reprod Health. 2019;51(1):7–15. 10.1363/psrh.12088. 30762937 PMC6476569

[10] Aiken AR Borrero S Callegari LS Dehlendorf C. Rethinking the pregnancy planning paradigm: unintended conceptions or unrepresentative concepts? Perspect Sex Reprod Health. 2016;48(3):147–151. 10.1363/48e10316. 27513444 PMC5028285

[11] Guide to DHS Statistics DHS-7. Current use of contraceptive methods. Accessed January 31, 2022. https://dhsprogram.com/data/Guide-to-DHS-Statistics/Current_Use_of_Contraceptive_Methods.htm

[12] Staveteig S. Fear, opposition, ambivalence, and omission: results from a follow-up study on unmet need for family planning in Ghana. PLoS One. 2017;12(7):PLoS One. 10.1371/journal.pone.0182076. 28759624 PMC5536298

[13] Upadhyay UD Dworkin SL Weitz TA Foster DG. Development and validation of a reproductive autonomy scale. Stud Fam Plann. 2014;45(1):19–41. 10.1111/j.1728-4465.2014.00374.x. 24615573

[14] Senderowicz L. Contraceptive autonomy: conceptions and measurement of a novel family planning indicator. Stud Fam Plann. 2020;51(2):161–176. 10.1111/sifp.12114. 32358789

[15] Moreau C Shankar M Helleringer S Becker S. Measuring unmet need for contraception as a point prevalence. BMJ Glob Health. 2019;4(4):e001581. 10.1136/bmjgh-2019-001581. 31543991 PMC6730575

[16] He K Dalton VK Zochowski MK Hall KS. Women's contraceptive preference-use mismatch. J Womens Health (Larchmt). 2017;26(6):692–701. 10.1089/jwh.2016.5807. 27710196 PMC5512313

